# Accelerated vs. conventionally fractionated postoperative radiotherapy of non-small cell lung cancer—final results of the prematurely terminated PORTAF trial

**DOI:** 10.1007/s00066-025-02422-y

**Published:** 2025-07-15

**Authors:** Rebecca Bütof, Lydia Koi, Steffen Löck, Steffen Appold, Steffen Drewes, Dirk Koschel, Jörg Kotzerke, Ursula Nestle, Sonja Adebahr, Daniel Zips, Frank Heinzelmann, Thomas Hehr, Dagmar Bucher, Jürgen Heide, Claus Belka, Farkhad Manapov, Ewa Wasilewska-Tesluk, Jochen Fleckenstein, Mechthild Krause, Esther G. C. Troost, Michael Baumann

**Affiliations:** 1 OncoRay – National Center for Radiation Research in Oncology, Faculty of Medicine and University Hospital Carl Gustav Carus, TUD Dresden University of Technology, Helmholtz-Zentrum Dresden-Rossendorf, Dresden, Germany; 2 Department of Radiotherapy and Radiation Oncology, Faculty of Medicine and University Hospital Carl Gustav Carus, TUD Dresden University of Technology, Dresden, Germany; 3 National Center for Tumor Diseases (NCT), NCT/UCC Dresden, a partnership between DKFZ, Faculty of Medicine and University Hospital Carl Gustav Carus, TUD Dresden University of Technology, and Helmholtz-Zentrum Dresden-Rossendorf (HZDR), Dresden, Germany; 4 Institute of Radiooncology – OncoRay, Helmholtz-Zentrum Dresden – Rossendorf, Dresden, Germany; 5 Partner Site Dresden, German Cancer Consortium (DKTK), Dresden, Germany; 6 Department of Thoracic Surgery, Fachkrankenhaus Coswig, Coswig, Germany; 7 Department of Internal Medicine and Pneumology, Fachkrankenhaus Coswig, Coswig, Germany; 8 Department of Nuclear Medicine, Faculty of Medicine and University Hospital Carl Gustav Carus, TUD Dresden University of Technology, Dresden, Germany; 9 University Medical Center Freiburg, Faculty of Medicine, Department of Radiation Oncology, University of Freiburg, Freiburg, Germany; 10 Department of Radiation Oncology, University of Tübingen, Tübingen, Germany; 11 Deptartment of Radiation Oncology, Marienhospital Stuttgart, Stuttgart, Germany; 12 Radiotherapy Harburg – Bergedorf, Hamburg, Germany; 13 Department of Radiation Oncology, LMU University Hospital of Munich, Munich, Germany; 14 Department of Oncology, University of Warmia and Mazury, Olsztyn, Poland; 15 Department of Radiotherapy and Radiation Oncology, Saarland University Medical Center, Homburg/Saar, Germany; 16 German Cancer Research Center (DKFZ), Heidelberg, Germany; 17 Medical Department I, Division of Pneumology, University Hospital Carl Gustav Carus, TU Dresden, Dresden, Germany; 18 Department of Radiation Oncology, Kliniken Maria Hilf, Moenchengladbach, Germany; 19 Bavarian Cancer Research Center (BZKF), Munich, Germany; 20 Radiotherapy Department, Warmian-Masurian Cancer Center of the Ministry of the Interior and Administration’s Hospital, Olsztyn, Poland

**Keywords:** Postoperative radiotherapy, Non-small-cell lung cancer, Fractionation, Overall treatment time, Positron-emission tomography

## Abstract

**Purpose:**

A prolonged overall treatment time (OTT) has been demonstrated to adversely affect the primary radiation therapy (RT) outcome in various solid tumors, including non-small cell lung cancer (NSCLC). Retrospective data from our group suggested an advantage of shorter OTT also for postoperative RT (PORT) in patients with NSCLC. The PORTAF trial (ClinicalTrials.gov: NCT02189967) was initiated to prospectively test this hypothesis.

**Methods:**

The multicenter prospective randomized phase II trial in patients with NSCLC investigated whether an accelerated schedule of PORT (7 fractions per week, 2 Gy per fraction, OTT 3.5–4 weeks) improved outcome compared to conventional fractionation (5 fractions per week, 2 Gy per fraction, OTT 5–6 weeks). Target volumes and total radiation doses were stratified in both treatment arms based on individual risk factors. Primary endpoint of the study was locoregional tumor control (LRTC) 36 months after PORT, with 154 patients to be included in each arm.

**Results:**

Due to slow accrual and changed indications for PORT, we prematurely closed the trial in 2019. Between 2014 and 2019, eight recruiting centers included 27 evaluable patients. An interim safety analysis performed for the first 21 patients showed nonsignificant differences regarding grade 3 toxicities between the treatment arms, thus not meeting the termination criteria. LRTC was not significantly different between accelerated (73%) and conventionally fractionated RT (92%; *p* = 0.535). Noteworthily, in 21 FDG-PET/CT restagings before RT, an unexpectedly high number of locoregional recurrences (*n* = 4) and distant metastases (*n* = 2) were seen, resulting in changed treatment intentions for these patients.

**Conclusion:**

The prematurely closed PORTAF trial did not find significant differences in 3‑year LRTC when comparing accelerated versus conventionally fractionated irradiation. The observed additional benefit of FDG-PET/CT restaging prior to PORT should be further investigated in a larger cohort to optimize patient selection and avoid unnecessary side-effects.

## Introduction

Non-small cell lung cancer (NSCLC) is one of the world’s most common malignant diseases [1, 2]. Despite the clinical introduction of new systemic treatment options and improvement of treatment techniques, the prognosis of patients is still poor, with an average overall survival of 5.5–15.7% after 5 years [1, 2]. In locoregionally confined NSCLC, surgical resection is the mainstay of treatment, and in specific situations, postoperative radiotherapy (PORT) is discussed afterwards [2].

A prolonged overall treatment time (OTT), i.e., the interval between the first and last radiation fractions, has been shown to adversely affect locoregional tumor control (LRTC) and overall survival in primary irradiation of several solid tumors, including non-small cell lung cancer (NSCLC) [3–7]. Investigations on the influence of the OTT of PORT in patients with NSCLC have usually only examined intervals between surgery and the start of radiotherapy. These retrospective studies did not provide evidence of a survival benefit for patients with shorter waiting periods between surgery and the start of radiotherapy [8, 9]. In contrast, a retrospective analysis of our own patient data suggested an advantage of shorter OTT of radiotherapy for patients with NSCLC in the adjuvant situation [9].

To shed more light on this issue, the aim of the present study was to prospectively investigate whether accelerated versus conventionally fractionated irradiation in the postoperative situation of patients with NSCLC would improve 3‑year LRTC. Secondary endpoints included the evaluation of quality of life, overall survival, occurrence of distant metastases, and acute and late radiation-induced side effects.

Two randomized clinical trials failed to show a benefit in terms of overall survival for pN2 resected patients [10, 11]. One possible explanation for this as well as for the lack of benefit of shorter waiting times between surgery and PORT may be the presence of undetected local recurrences or distant metastases [12, 13]. Therefore, we decided to preferably use FDG-PET/CT restaging before irradiation in the PORTAF trial to have the best possible chance of identifying metastatic disease and to further evaluate the number of undetected locoregional recurrences.

## Methods and trial design

We here report the final results of the multicenter prospective randomized phase II PORTAF trial (ClinicalTrials.gov: NCT02189967) investigating whether accelerated PORT (7 fractions per week, 2 Gy per fraction) improved LRTC compared to conventional fractionation (5 fractions per week, 2 Gy per fraction) in patients with NSCLC. We used the CONSORT reporting guidelines.

### Recruitment, randomization, and workflow

The study protocol and associated procedures have been published previously [14]. In brief, all patients with signed informed consent were included in the study. Before randomization blood analyses and postoperative pulmonary function test needed to be done as well as a complete staging including FDG-PET/CT or, alternatively, contrast-enhanced CT of the chest/abdomen and bone scintigraphy and cerebral MRI in cases of suspected brain metastases. For patients receiving postoperative adjuvant chemotherapy, these restaging procedures had to be repeated prior to the start of radiotherapy.

**Fig. 1:**
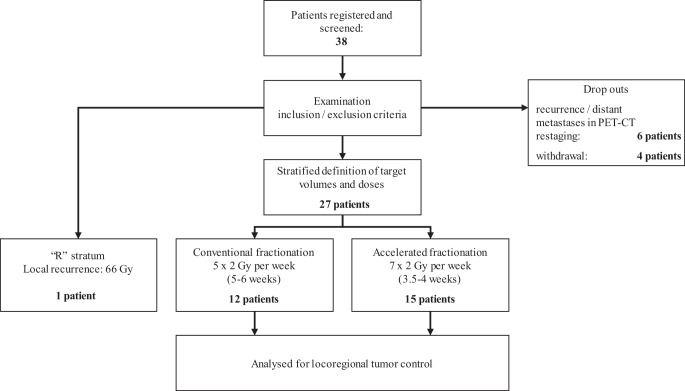
Flowchart of the study procedures

For electronic data collection and randomization, the RadPlanBio platform [15] was used by each study site, with stratification of patients by the following criteria: staging with or without PET/CT, resection status R1 or R0, and the respective study center. Patients with macroscopic tumor detected in pre-irradiation staging were treated in stratum “R” of the trial and received conventionally fractionated radiation therapy to a total dose of 66 Gy. Figure 1 shows a flowchart of the study and Table 1 the inclusion and exclusion criteria.

**Table 1:** Inclusion and exclusion criteria

Inclusion criteria	Exclusion criteria
Histologically confirmed non-small cell lung cancer	Histologically confirmed small cell lung cancer
Surgery performed with curative intent	Distant metastases (M1)
Postoperative indication for radiotherapy (> pN1 and/or R1)	Patient is unable to understand reason, purpose, and potential side effects of the study
Local recurrence postoperatively/after adjuvant chemotherapy diagnosed by the restaging imaging (“R” stratum)	Previous (< 5 years) or synchronous malignancy (except in situ carcinomas, basal cell carcinoma, early-stage skin cancer)
No distant metastases (M0)	Previous radiotherapy in the thoracic or lower head and neck region
Patient ≥ 18 years old	Pregnancy or lactation
Good general health condition (ECOG score 0 or 1)	Participation in another clinical intervention trial or not completed follow-up of a clinical intervention study (except psychological, supportive, or observational studies)
Signed informed consent	
Appropriate compliance to ensure follow-up visits	
Women of childbearing age: adequate contraception	

### Primary and secondary endpoints

The primary endpoint of this study was locoregional tumor control 36 months after the start of radiotherapy comparing an accelerated (7 fractions per week, 2‑Gy single dose) with the standard conventionally fractionated schedule (5 fractions per week, 2‑Gy single dose) for postoperative radiotherapy in patients with NSCLC.

Locoregional tumor control was defined as freedom from local recurrence and regional recurrence (in-field, at the edge of the field, or out of field). Secondary endpoints included overall survival, local recurrence-free and distant metastases-free survival 36 months after the start of radiotherapy, acute and late toxicity, and quality of life for both treatment methods.

Clinical follow-up examinations with imaging scans (FDG-PET/CT or CT of the thorax/abdomen) were scheduled every 3 months within the first 3 years and every 6 months for the following 2 years. During radiation therapy and at the follow-up visits, scoring of side effects was performed according to the Common Terminology Criteria for Adverse Events (CTC-AE) 4.0. Quality of life was assessed additionally by the European Organisation For Reserach and Treatment of Cancer (EORTC) questionnaires QLQ-C30 and QLQ-LC13 before and at the end of irradiation and at each follow-up visit.

The trial design and protocol adhere to the SPIRIT criteria (Standard Protocol Items: Recommendations for Interventional Trials; www.spirit-statement.org) [14].

### Biometric design

Target volumes and total radiation doses were stratified in both treatment arms based on individual risk factors. The initial sample size calculation was based on the hypothesis of an improvement of 15% in the primary endpoint, i.e., an increase in the locoregional tumor control rate from 70 to 85% after 3 years in the accelerated treatment arm (7 fractions per week) in the group of R0- or R1-resected patients. Assuming α to be 0.05, a rise of 15% could be detected with a power of 80% if 154 patients were to be treated in each arm. This number includes a dropout rate of 10%.

The observational “R” stratum, which contains patients with macroscopic remaining tumor or recurrences detected in pre-irradiation staging, was not included in the statistical sample size calculation.

### Radiotherapy

For radiation planning purposes, all patients should preferably have undergone FDG-PET/CT in the radiation treatment position. On the basis of the planning CT, the clinical target volume and the organs at risk were contoured in accordance with the trial protocol. Standardization of contouring was achieved by a mandatory dummy run for all participating centers with quality checks by three experienced radiation oncologists (SA, RB, ET). Each patient was treated with an image-guided conformal radiation technique (3D-CRT; intensity-modulated RT, IMRT).

The mediastinal target volume received a total dose of 50 Gy, and in the case of microscopic incomplete resection (R1) or extracapsular tumor spread (ECE), additional boost irradiation of 10 Gy (2 Gy per fraction) to a total dose of 60 Gy was delivered. Accelerated radiotherapy was conducted by application of a second fraction on two weekdays and/or an additional irradiation at weekends. It was mandatory to have an interval of at least 6 h between treatment fractions, preferably 8 h.

### Data monitoring committee

The Data Monitoring Committee (DMC) consisted of five members: four clinicians with experience in the study-specific indication area of radiotherapy (V. Budach, R. D. Kortmann, R. Schwarz, D. Vordermark) and one biometrician (P. Martus). The members of the DMC were independent from the study centers and have declared that there exists no scientific or financial conflict of interest. The DMC reviewed the results of safety analyses and quality controls as well as the evaluations of toxicities and their impact on the benefit–risk assessment for the patients twice.

## Results

In total, eight recruiting centers included 27 evaluable patients (additional 10 dropouts and one stratum “R” patient) into this trial between 2014 and 2019. Due to slow accrual and altered indications for postoperative radiotherapy, we prematurely closed this trial on recommendation by the DMC in 2019.

**Fig. 2:**
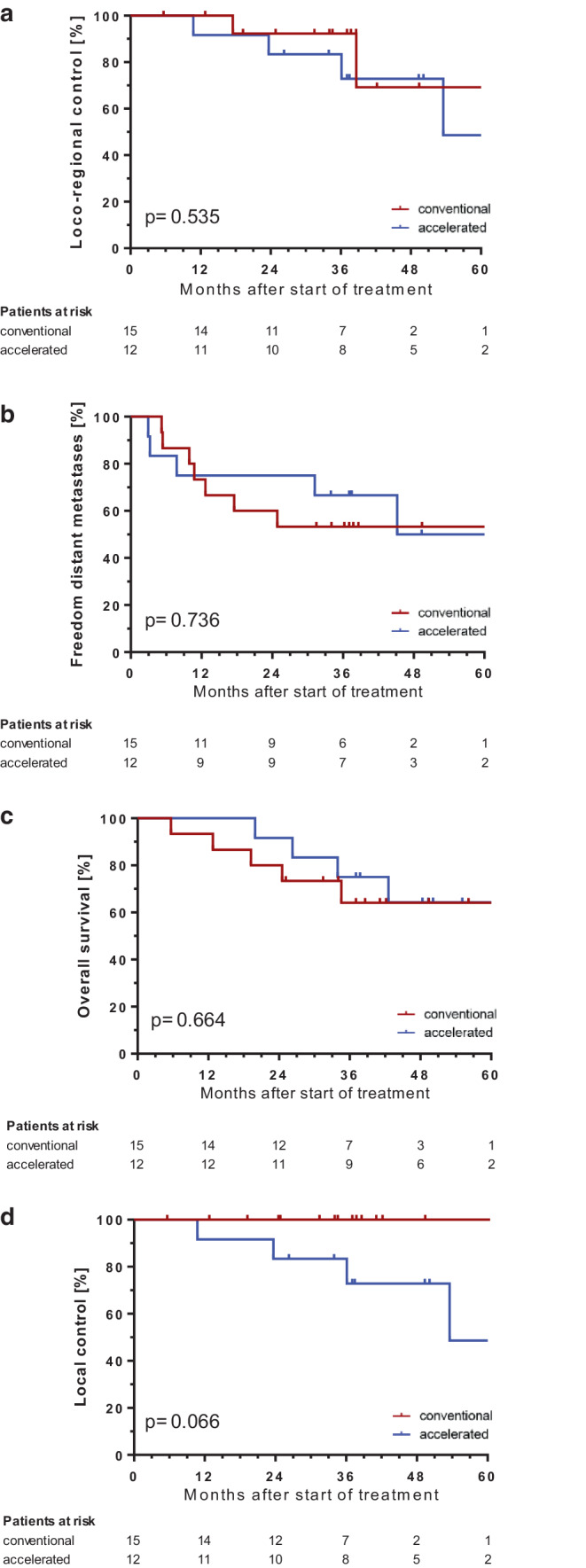
Kaplan–Meier curves of locoregional tumor control as the primary endpoint (**a**) as well as local control, freedom from distant metastases, and overall survival as secondary endpoints (**b**, **c**, **d**) comparing conventional vs. accelerated fractionation and corresponding *p*-values

In the accelerated treatment group, 9 patients received 50 Gy and 3 patients 60 Gy, whereas in the conventional treatment group, 7 patients were given 50 Gy and 8 patients 60 Gy (Table 2). Locoregional tumor control as the primary endpoint at 3 years was not significantly different after accelerated (73%) versus conventional irradiation (92%; HR 1.682, 95% CI [0.337; 8.37]; *p* = 0.535; Fig. 2). Due to the low statistical power, a final conclusion on the primary objective is not possible. Overall survival at 5 years was identical (64% vs. 64%; *p* = 0.664), and freedom from distant metastases was similar (50% vs. 53%; *p* = 0.736; Fig. 2). Overall, nine patients died during the observation period (eight tumor-related deaths and one unknown cause of death).

**Table 2:** Patient and treatment characteristics comparing the conventional and accelerated groups

Characteristic	Conventional (*n* = 15)	Accelerated (*n* = 12)
*Age (years; median [range])*	60 [39–73] (*n* = 7)	68 [59–75] (*n* = 6)
*T status (n)*		
pT1	2	3
pT2	7	6
pT3	1	1
pT4	5	2
*N status (n)*		
pN0	0	1
pN1	1	0
pN2	13	11
pN3	1	0
*M status (n)*		
cM0	15	12
*R status (n)*		
R0	11	11
R1	4	1
*Grading (n)*		
G2	6	7
G3	8	5
Gx	1	0
*Adjuvant chemotherapy*		
yes/no	11/4	10/2
*Applied dose*		
50 Gy	7	9
60 Gy	8	3

An interim safety analysis was performed per protocol in August 2018 for the first 21 patients with a minimum follow-up of 3 months: no grade 4 or 5 events occurred up to this timepoint. Furthermore, no deviation of grade ≥ 3 events between conventional and accelerated fractionation was found (Table 3), thus not meeting the termination criteria. Further safety analyses were not done, since the necessary number of included patients had not been reached. During the observation period, no grade 4 or 5 toxicities were reported.

**Table 3:** Results of the interim safety analysis for the first 21 patients

Toxicity grade 3	Conventional fractionation	Accelerated fractionation
Pain	1 event at follow-up visit 6 weeks after irradiation	1 event in week 1 of treatment
Dysphagia	–	1 event in week 1 of treatment
		1 event in week 4 of treatment
Dyspnea	–	1 event in week 4 of treatment
Cough	1 event at follow-up visit 6 weeks after irradiation	–

Of 27 evaluable patients, six were treated with surgery only and 21 received adjuvant chemotherapy after surgery (Table 2). Additionally, there was a high number of dropout patients (*n* = 10; 26%). Noteworthily, 6 out of 21 restaging FDG-PET/CTs before radiotherapy revealed unexpected extensive local and regional recurrences (*n* = 4) or distant metastases (*n* = 2), resulting in completely changed treatment intentions for these patients from a curative setting to palliative systemic treatment. Five of these patients had received adjuvant chemotherapy before, one had only surgery. There were no differences in the time intervals between surgery and PET imaging for patients with and without unexpected progression receiving adjuvant chemotherapy (135 vs. 140 days). In addition, for patients with surgery only, these time intervals have also been equal in both groups with and without progression in PET restaging (47 vs. 45 days).

**Fig. 3:**
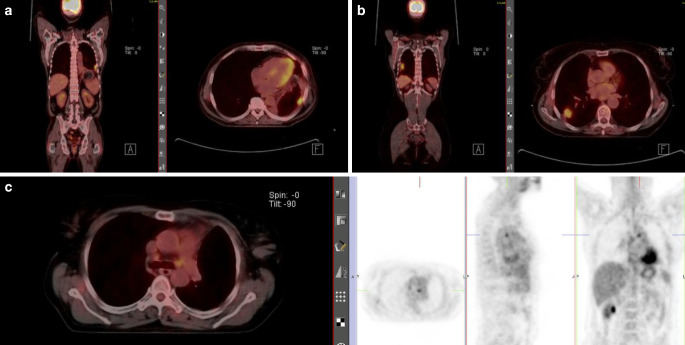
FDG-PET/CT restaging of three exemplary patients with formerly undetected metastatic disease or local recurrence: **a** male patient with histologically proven pleural metastasis after adjuvant chemotherapy; **b** female patient with local recurrence after adjuvant chemotherapy, included in stratum “R” of the trial; and **c** female patient with lymph node metastasis in regio 4L after adjuvant chemotherapy

One patient with a local recurrence was included in the “R” stratum of the trial, treated with 66 Gy in conventional fractionation as a curatively intended dose. Figure 3 shows the FDG-PET/CT restaging of three exemplary patients with formerly undetected metastatic disease or local recurrence.

## Discussion

The PORTAF phase II randomized trial was designed to test whether an accelerated postoperative irradiation schedule in patients with NSCLC resulted in higher locoregional tumor control rates than conventional fractionation. This hypothesis was based on our retrospective study, which highlighted the overall treatment time of postoperative radiotherapy as an important factor for adverse LRTC rates [9]. This OTT effect has also been demonstrated in randomized trials and meta-analyses of primary radiotherapy of NSCLC [3–7]. As the main radiobiological mechanism of this phenomenon, (accelerated) repopulation of tumor cells during the course of radiotherapy is considered. Some observations suggest that chemotherapy applied before radiotherapy may potentially enhance repopulation, and chemotherapy, in turn, is frequently applied before PORT [16].

The PORTAF trial was prematurely closed mainly due to slow accrual and altered clinical indications for postoperative radiotherapy after two negative studies on overall survival in pN2 resected patients [10, 11]. Our results show neither a difference between the treatment arms for the primary endpoint, nor for the secondary endpoints. However, the most important limitation of this study is the low number of patients included. Therefore, due to lacking statistical power, no final conclusions regarding the effectiveness of the treatment schedules of the PORTAF trial are feasible. For illustration, a difference of approximately 60% in locoregional control would be required to detect a significant difference between the treatment arms with a power of 80% based on the 15 and 12 patients recruited.

The ongoing debate on indications for postoperative irradiation based on former literature and the two recent randomized trials for pN2 patients with negative study results underscore the need for better selection of high-risk patients who might benefit from PORT [10, 11].

The randomized PORT‑C trial [10] reported on 310 completely resected pIIIA-N2 stage NSCLC patients treated per protocol out of 394 included in the study and found a statistically significant increase in disease-free survival (DFS) for PORT compared to observation, without resulting in better overall survival rates (*p* = 0.41). The subsequent intention-to-treat analysis did not reveal a difference in DFS (*p* = 0.20). Similar results were obtained in the LungART trial [11] published in 2021: DFS and OS were not statistically significantly different between PORT and the observation group. Nevertheless, in the LungART trial, a significantly reduced risk of locoregional mediastinal recurrence was reported after PORT. These results highlight the necessity of improved patient selection for individualized indications for radiotherapy in order to achieve clinically relevant benefits, e.g., in high-risk situations with extracapsular extension in multiple involved lymph nodes or after R1 resection. One possible explanation for the two negative trials as well as for the lack of benefit of shorter waiting times between surgery and PORT in our own retrospective evaluation may be undetected local recurrences or distant metastases [12, 13]. Therefore, we decided to preferably use FDG-PET/CT restaging before irradiation within our trial to identify metastatic disease and locoregional recurrences.

In the PORTAF trial, we observed a high proportion of patients (29%) with local and regional recurrences or with distant metastases in FDG-PET/CT restaging after surgery alone or after surgery and adjuvant chemotherapy. Noteworthily, the recent EANM/SNMMI/ESTRO recommendation on the use of FDG-PET/CT in (N)SCLC patients did not elaborate on the use of FDG-PET in the postoperative setting, since data are missing [17]. Thus, the standard imaging modality acquired prior to initiation of PORT is whole-body CT with intravenous contrast agent, which, in our study, did not reveal any recurrences or distant metastases.

The fact that almost 30% of patients had locoregional or distant progression may well be one possible explanation for the missing clinical benefit of radiotherapy in this and the aforementioned cohorts, since patients with metastatic disease would otherwise be excluded due to the palliative situation. Therefore, we hypothesize that regular FDG-PET/CT restaging after surgery or adjuvant chemotherapy prior to PORT might be useful to detect unexpected metastatic disease. If our results were to be proven in a larger patient cohort, this could on the one hand significantly increase the clinical benefit of postoperative irradiation and optimize patient selection, and on the other hand patients’ toxicity burden could be reduced by avoiding unnecessary treatment.

The most important limitation of this prematurely closed study was slow accrual, resulting in a low number of patients included. Therefore, the final comparison of the two treatment schedules remains inconclusive. Additionally, the majority but not all patients received PET-CT restaging, which could limit the results.

## Conclusion

The prematurely closed PORTAF trial did not find significant differences in 3‑year locoregional tumor control when comparing accelerated versus conventionally fractionated irradiation. The observed additional benefit of FDG-PET/CT restaging prior to PORT should be further investigated in a larger cohort to optimize patient selection and avoid unnecessary side effects.
